# Annotated Bioinformatic Pipelines for Genome Assembly and Annotation of Mitochondrial Genomes

**DOI:** 10.21769/BioProtoc.5231

**Published:** 2025-03-05

**Authors:** Jessica C. Winn, Aletta E. Bester-Van Der Merwe, Simo N. Maduna

**Affiliations:** 1 Molecular Breeding and Biodiversity Group, Department of Genetics, Stellenbosch University, Stellenbosch, Western Cape, South Africa; 2 Department of Ecosystems in the Barents Region, Svanhovd Research Station, Norwegian Institute of Bioeconomy Research, Svanvik, Norway

**Keywords:** Comparative mitogenomics, Hybrid assembly, Intramitochondrial recombination, Next-generation sequencing

## Abstract

Mitochondrial genomes (mitogenomes) display relatively rapid mutation rates, low sequence recombination, high copy numbers, and maternal inheritance patterns, rendering them valuable blueprints for mapping lineages, uncovering historical migration patterns, understanding intraspecific population dynamics, and investigating how environmental pressures shape traits underpinned by genetic variation. Here, we present the bioinformatic pipeline and code used to assemble and annotate the complete mitogenomes of five houndsharks (Chondrichthyes: Triakidae) and compare them to the mitogenomes of other closely related species. We demonstrate the value of a combined assembly approach for detecting deviations in mitogenome structure and describe how to select an assembly approach that best suits the sequencing data. The datasets required to run our analyses are available on the GitHub and Dryad repositories.

Key features

• Tips and code for conducting de novo, reference-based, and hybrid assembly.

• Guide to detecting deviations in the structure of the mitochondrial genome.

• Step-by-step guide to annotating and comparing the characteristics of mitochondrial genomes.

• Access to the scripts, data files, and pipelines used to enable replication of all analyses.

## Graphical overview


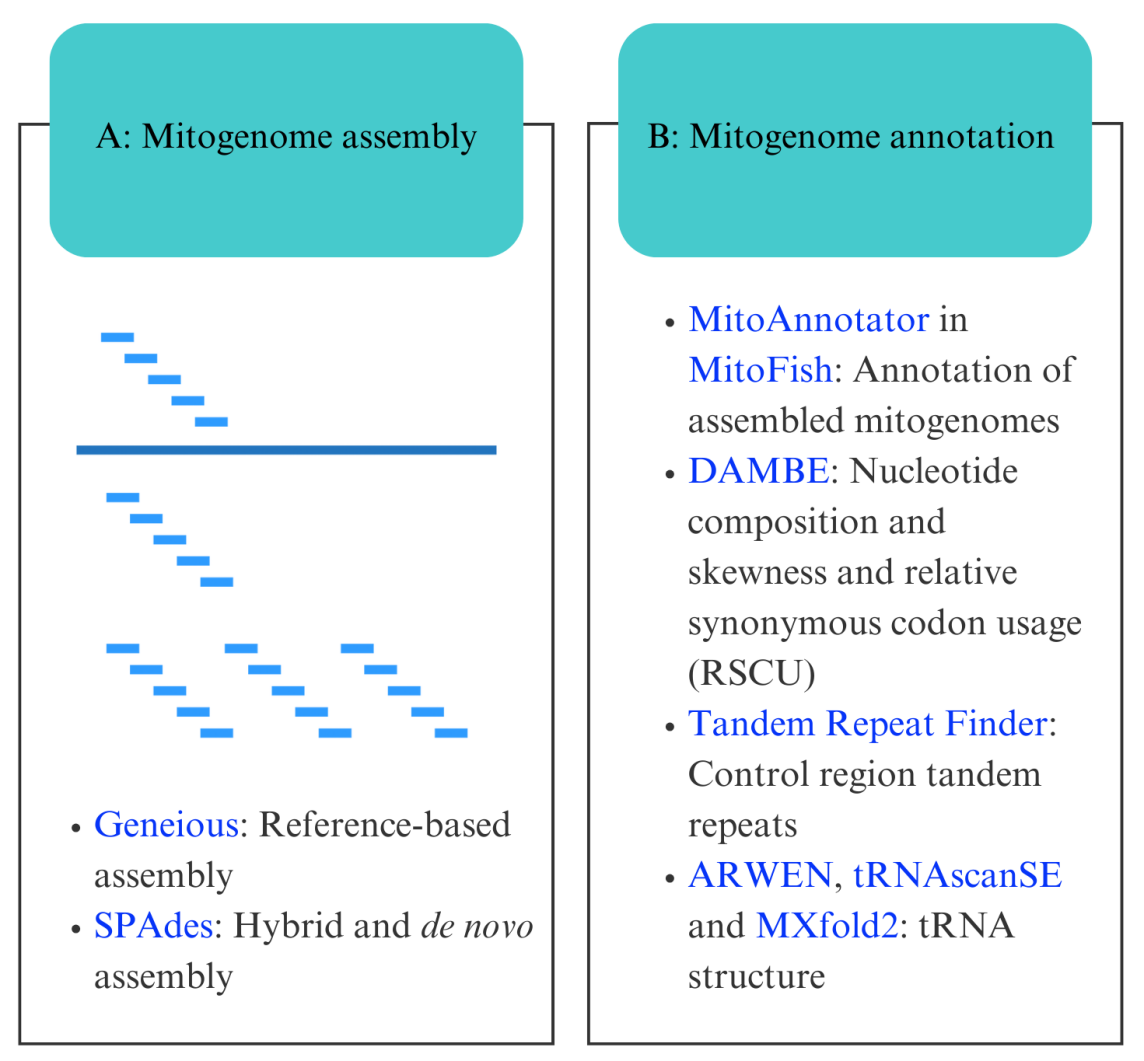


**Bioinformatic workflow for mitogenome assembly and annotation.** Software programs are indicated in blue.

## Background

Mitochondrial genomes (mitogenomes) are found in high copy numbers in the majority of eukaryotic cells and are among the most abundant genome sequences deposited in nucleotide databases [1,2]. In animals, the mitogenome ranges from 14 to over 20 kilobase pairs (kbp) in size and, although gene order can vary, gene content is highly conserved [3,4]. Mitogenomes contain short intergenic spacers, a limited occurrence of gene duplications and rearrangements, and generally lack introns [4,5]. These features make them ideal candidates for investigating complex evolutionary processes at a resolution that far surpasses that of nuclear genomes [4]. Despite gene content being conserved in most species, gene arrangements and duplications can distinguish specific evolutionary lineages, making them useful for resolving taxonomic relationships, conducting population genomics investigations, and contributing to environmental DNA metabarcoding databases [6–8]. Single mitochondrial genes have commonly been used to assess interspecific divergence and intraspecific diversity; however, their phylogenetic signal is transcended by that of the full mitogenome, making mitogenomic analyses more powerful as well as comparable across a wider range of studies [6,9–11].

Genomic studies are being revolutionised by the increasing availability of next-generation sequencing (NGS) technologies, which have enabled the procurement of mitogenomes rapidly and at a low cost [12,13]. However, there is a shortage of standardised pipelines for the full reconstruction and characterisation of these small molecules [14]. The assembly of mitogenomes from high-throughput sequencing (HTS) data can be achieved by mapping reads to a reference mitogenome or by using de novo assembly. Tools such as the Burrows-Wheeler Alignment tool [15], Bowtie2 [16], Minimap2 [17], BBMap [18], and Geneious Prime [19] (https://www.geneious.com/download, last accessed 1/12/2025) can be used for reference-based assembly. This is the less computationally intensive approach; however, it relies on the use of a closely related, well-annotated reference mitogenome to avoid erroneous reconstruction. It may also collapse duplicated regions and fail to detect structural deviations from the reference, particularly when utilizing short reads generated by Illumina or Ion Torrent platforms [20,21]. The alternative approach is a de novo assembly using tools such as SPAdes [22], MitoZ [23], MITObim [24], MitoHiFi [25], and NOVOPlasty [26], which are useful in the absence of a closely related reference or for a contiguous assembly of complex, repetitive, or structurally variable regions of the mitogenome with short reads [21]. Long-read sequencing technologies, including PacBio HiFi sequencing and Oxford Nanopore Technology (ONT) sequencing, circumvent many of these limitations [27,28]. However, a carefully designed assembly approach can ensure a successful assembly of more complex mitogenomes with short-read sequences from Illumina and Ion Torrent platforms.

In this protocol, we describe a three-step mitogenome assembly approach and provide a guide for detecting structural deviations using sequencing data generated for five species belonging to the Triakidae family (houndsharks; Linck 1790 [29]) in Section A. Section B provides a detailed annotation and comparative mitogenomics pipeline. This protocol can be used to design mitogenomic assembly pipelines and serves as educational material for various higher-education modules in molecular evolution.

## Software and datasets

Most of the software programs listed below can be used on Windows 7/8/10/11, Mac OS 10.11 (current versions), and Linux (Ubuntu Desktop LTS, last two supported versions). A 64-bit Linux system or Mac OS (with Python 2.7 and Python 3: 3.2 and higher to be pre-installed on it) is required to run SPAdes and Quast. If you do not have Linux on your device, you can use MobaXTerm v.24.4 (https://mobaxterm.mobatek.net/download.html, last accessed 1/12/2025) for Windows or Tabby Terminal v.1.0.216 (https://tabby.sh/, last accessed 1/12/2025) for Mac to run a command line code.

We used a machine with a multi-core central processing unit (CPU) allowing for parallel processing to speed up some of our analyses. The amount of RAM depends on the size of the dataset, but at least 8 GB is recommended. If high-performance computing (HPC) resources are not available, the CIPRES (Cyberinfrastructure for Phylogenetic Research) Science Gateway portal v.3.3 at the San Diego Supercomputer Centre [30] (https://www.phylo.org/, last accessed 1/12/2025) is an online platform that provides a user-friendly web interface for performing computationally intensive phylogenetic analyses. SPAdes can be run through CIPRES, and there is a Geneious plugin too. Users can subscribe to a three-month free trial with 1,000 CPU hours; thereafter, CPU hours can be purchased.

**A. Mitogenome assembly**

1. FastQC version (v).12.0 (http://www.bioinformatics.babraham.ac.uk/projects/fastqc/, last accessed 1/12/2025) for quality control of sequencing files. It requires a suitable Java runtime environment (https://adoptium.net/, last accessed 1/12/2025)

2. Geneious Prime v.2023.2 [19] (https://www.geneious.com/download/, last accessed 1/12/2025) for reference-based assembly and assembly editing and comparison. Note that although reference assembly using the Geneious assembly tool is available in the free version, a paid license is required to edit assemblies or alignments. Mega11 [31] (https://www.megasoftware.net/, last accessed 1/12/2025) is an alternative software that can be used for sequence visualisation and editing. Geneious also provides licenses for students doing courses (see https://www.geneious.com/free-course-license, last accessed 1/12/2025). Alternatively, there are other programs available for reference-based mitogenome assembly (e.g., GetOrganelle [32] and MITObim [24]). The Burrows-Wheeler Alignment tool [15] can also be used to extract mitogenome reads via alignment to a reference, which can then be assembled in SPAdes. Remember to select the software most suited to your organism under study

3. SPAdes v.3.15 [22] (https://github.com/ablab/spades, last accessed 1/12/2025) for hybrid and de novo assembly. SPAdes can now be run in the latest version of Geneious. When you run the SPAdes assembler for the first time, Geneious provides instructions for installing the necessary Windows or Mac features onto your device

4. Quast v.5.0.2 [33] (https://github.com/ablab/quast/releases, last accessed 1/12/2025) for assembly quality checking

5. FinchTV 1.5 (https://digitalworldbiology.com/FinchTV, last accessed 1/12/2025) for sequence trimming for Sanger sequences

6. Our raw Ion Torrent bam files for mitogenome assembly are available on the SRA database (BioProject repository):

a. Data identification number: PRJNA997468 (BioSample accessions: SAMN36680060, SAMN36680061, SAMN36680062, SAMN36680063, SAMN36680064)

b. Direct URL to data (last accessed 1/12/2025): https://www.ncbi.nlm.nih.gov/bioproject/997468; https://www.ncbi.nlm.nih.gov/biosample/36680060; https://www.ncbi.nlm.nih.gov/biosample/36680061; https://www.ncbi.nlm.nih.gov/biosample/36680062; https://www.ncbi.nlm.nih.gov/biosample/36680063; https://www.ncbi.nlm.nih.gov/biosample/36680064

c. Alternatively, filtered reads for the mitogenome can be found in the folder *1_Raw_Ion Torrent_NGS_data: Data_1_Galeorhinus_galeus_Ion Torrent_Filtered_RawData, Data_2_Mustelus_asterias_Ion Torrent_Filtered_RawData, Data_3_Mustelus_mosis_Ion Torrent_Filtered_RawData, Data_4_Mustelus_palumbes_Ion Torrent_Filtered_RawData, Data_5_Triakis_megalopterus_Ion Torrent_Filtered_RawData* on the Dryad Digital Repository (doi: 10.5061/dryad.sj3tx969h, last accessed 1/12/2025)

**B. Mitogenome annotation**

1. MitoAnnotator in MitoFish v.3.72 webserver [34,35] (http://mitofish.aori.u-tokyo.ac.jp/annotation/input/, last accessed 1/12/2025) for mitogenome annotation

2. Sequence Manipulation Suite 2 [36] (https://www.bioinformatics.org/sms2/translate.html, last accessed 1/12/2025) for checking the reading frame of protein-coding genes

3. GenBank [37] submission platform (https://www.ncbi.nlm.nih.gov/WebSub/, last accessed 1/12/2025) for submission of GenBank files

4. DAMBE v.7.0.35 [38,39] (http://dambe.bio.uottawa.ca/DAMBE/dambe.aspx, last accessed 1/12/2025) for calculating nucleotide composition and skewness and relative synonymous codon usage

5. R (https://cran.r-project.org/, last accessed 1/12/2025) and R Studio (https://posit.co/products/open-source/rstudio/, last accessed 1/12/2025) for constructing nucleotide composition, skewness, and RSCU plots

6. ARWEN v.1.2.3 webserver [40] (http://130.235.244.92/ARWEN/, last accessed 1/12/2025), tRNAscanSE webserver v.2.0 [41] (http://lowelab.ucsc.edu/cgi-bin/tRNAscan-SE2.cgi, last accessed 1/12/2025), and MXfold2 [42] webserver (http://ws.sato-lab.org/mxfold2/, last accessed 1/12/2025) for checking tRNA folding

7. Tandem Repeat Finder [43] (https://tandem.bu.edu/trf/trf.html, last accessed 1/12/2025) for characterising repeats in the control region

8. Mitogenome sequence files for our Triakidae mitogenomes can be found on GenBank. Data identification numbers: ON075075, ON075076, ON075077, ON652873, and ON652874. Direct URL to data (last accessed 1/12/2025): https://www.ncbi.nlm.nih.gov/nuccore/ON075075 ; https://www.ncbi.nlm.nih.gov/nuccore/ON075076 ; https://www.ncbi.nlm.nih.gov/nuccore/ON075077 ; https://www.ncbi.nlm.nih.gov/nuccore/ON652873 ; https://www.ncbi.nlm.nih.gov/nuccore/ON652874

a. Alternatively, these have been uploaded to the folder *2_Galeomorphii_mitogenome_sequences: Data_8_ON652873_Mustelus_asterias, Data_9_ON652874_Galeorhinus_galeus, Data_10_ON075075_Triakis_megalopterus, Data_11_ON075076_Mustelus_palumbes, Data_12_ON075077_Mustelus_mosis* in on our Dryad Digital Repository ( doi: 10.5061/dryad.sj3tx969h , last accessed 1/12/2025)

## Procedure

**A. Mitogenome assembly**

The mitogenome assembly and annotation pipeline was used to assemble Ion Torrent sequencing reads in bam format in a three-step approach to detect deviations in mitogenome structure. Although the pipeline here is demonstrated using Ion Torrent unpaired reads, it will also work with paired-end/high-quality mate-paired Ion Torrent reads, Illumina paired-end/high-quality mate-paired/unpaired reads, and PacBio Circular Consensus Sequencing (CCS) reads [22]. Refer to the SPAdes manual (https://home.cc.umanitoba.ca/~psgendb/doc/spades/manual.html) for input format requirements and k-mer adjustments for different read types. SPAdes works by progressively refining the assembly by iterating through De Bruijn graphs of increasing k-mer sizes. This allows the assembler to balance between smaller k-mers, which provide better coverage and connection across repetitive regions, and larger k-mers, which increase specificity and help resolve repeats and reduce ambiguity [22]. The graphs from different k-mer sizes are then merged and used for assembly. This approach is best suited for small to medium genomes and shorter sequencing reads. Alternative programs utilizing Overlap-Layout-Consensus (OLC) assembly are preferable for more complex genomes and long sequencing reads, which include PacBio HiFi and Oxford Nanopore Technologies (ONT) reads.

Raw reads are used as input files for de novo and reference-based assembly to a high-quality assembled mitogenome of a closely related species. We also developed a hybrid assembly approach that improves on the base call ambiguities and length biases of reference assembly with short reads while reducing the computational requirements that come with de novo assembly. Nevertheless, we show that for identifying and characterising large structural rearrangements, a de novo assembly is beneficial. Large duplications and/or rearrangements can be confirmed with a basic Sanger sequencing approach. The hybrid assembly approach can help detect when there is a region that could be investigated further with de novo assembly. However, in cases where a region is duplicated more than once with minor differences between the duplicates, short reads, generated with Ion Torrent and Illumina sequencing, may still cluster together to form one consensus sequence, revealing only one duplicate. Long-read technologies such as PacBio and Nanopore sequencing would be useful for addressing this challenge. SPAdes is able to assemble short reads with long reads to close gaps and resolve repeats if you have additional long-read sequences available.

The read depth required for accurate mitogenome assembly depends on the type of analysis being conducted, as different applications demand varying levels of coverage [44]. A minimum read depth of 15× is cited as suitable for whole-genome assembly with Ion Torrent reads for non-clinical applications [13]. However, it is important to note that read depth will vary across the assembly. Read depth varied from 4× to 154× in our assemblies. *Galeorhinus galeus* (ON652874) is used as the example mitogenome for this protocol, and *Mustelus mustelus* (NC_039629.1 [45]) is used as the reference mitogenome for reference-based assembly. Replace these species identifiers with your own species ID when using the pipeline for your own samples.

**Figure 1.**
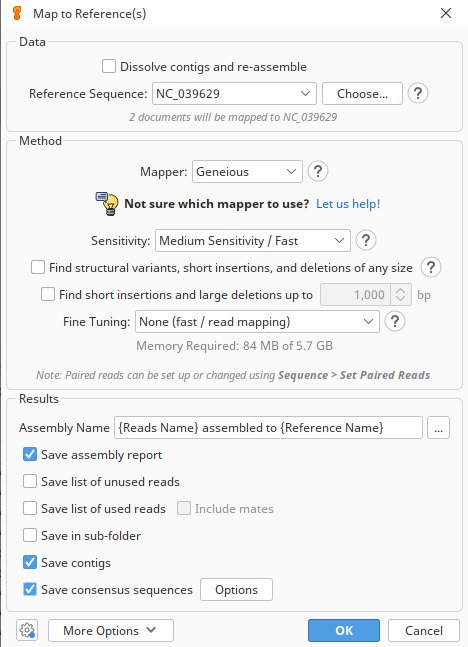
Parameters used for the reference-based assembly of *Galeorhinus galeus* Ion Torrent bam reads. Reads were mapped to the mitogenome of *Mustelus mustelus* (NC_039629 [45]) in Geneious Prime v.2024.0.2 [19] using medium sensitivity settings and five iterations; all other parameters were left at their default settings.

1. Reference-based assembly

a. Import the raw sequencing reads in bam format [our raw Ion Torrent bam files are available on the SRA database (BioProject PRJNA997468)] and the representative reference mitogenome of a closely related species in GenBank format to Geneious. This can be retrieved from the nucleotide database on NCBI (https://www.ncbi.nlm.nih.gov/nucleotide/).

b. Assemble the reads to the reference mitogenome using the Geneious read mapper with medium sensitivity settings and five iterations (Figure 1). These are the default settings for the Geneious read mapper and, for most situations, using the default sensitivity is recommended.

The highest sensitivity is intended for use with smaller numbers of Sanger reads (1,000 or less), and medium or medium-low sensitivity is usually the best option for large numbers (e.g., 100,000 or more) of next-generation sequencing reads [19]. Iterative fine-tuning maps reads to the consensus sequence from the previous iteration and converts the reads back to mappings relative to the reference sequence, repeating the process until the maximum number of iterations is reached. Iterative assembly greatly improves results around regions that differ from the reference sequence. Later iterations generally map a higher fraction of reads as the mapping extends into regions where reads were previously un-mappable. Geneious recommends using five iterations. Decreasing this will increase the assembly speed.

**Figure 2.**
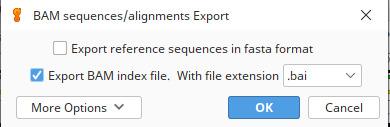
Selections for saving reads in bam format in Geneious Prime v.2024.0.2 [19]. These filtered reads that mapped to a reference mitogenome can now be used as input for de novo assembly (hybrid assembly approach).

2. Hybrid assembly

a. In Geneious, select your reference assembly, click *Export | Export Documents*, and choose to save the file in bam format by selecting *Export BAM index file* (Figure 2).

b. Feed the bam reads into a de novo pipeline in SPAdes with the input set for unpaired Ion Torrent reads with eight threads, k-mers 21,33,55,77,99,127, the careful option to reduce the number of mismatches and short indels, and all other parameters left as default. For datasets with high GC content or low or uneven coverage, consider enabling the single-cell mode (setting –sc option) and use k-mer lengths of 21,33,55. Shorter k-mers can help mitigate the increased sequencing error rate that often occurs in these cases. Use Quast to generate an assembly report. If you are running SPAdes in Geneious, select your reference assembly and click *Align/Assemble | De Novo Assemble*. Select *SPAdes* as your assembler. You can also paste these parameters in the *Custom SPAdes Options* bar.

work = ./Spades_hybrid_assembly

spades.py \

-k 21,33,55,77,99,127 \

-t 8 \

-m 250 \

--Ion Torrent \

-s $work/Data_1_Galeorhinus_galeus_Ion Torrent_Filtered_RawData.bam \

--careful \

-o $work/G_galeus_Hybrid_Assembly \

quast.py \

-o $work/G_galeus_Hybrid_Assembly/Quast_G_galeus \

$work/G_galeus_Hybrid_Assembly/contigs.fasta \

3. De novo assembly

a. Directly map the raw reads (in bam format) de novo and generate an assembly report. See step A2b above for k-mer setting adjustments.

work = ./Spades_de_novo_assembly

spades.py \

-k 21,33,55,77,99,127 \

-t 8 \

-m 250 \

--Ion Torrent \

-s $work/IonCode_G_galeus_rawlib.basecaller.bam \

--careful \

-o $work/G_galeus_Denovo_Assembly \

quast.py \

-o $work/G_galeus_Denovo_Assembly/Quast_G_galeus \

$work/G_galeus_Denovo_Assembly/contigs.fasta \

4. Assembly comparison

a. Open the *Assembly Report* file produced during assembly in Geneious in step A1 and note the number of reads that mapped to the reference mitogenome and the size of the contigs produced. Open the Quast files titled *report.pdf* in the de novo and hybrid assembly folders from steps A2 and A3 above. Note the total number of contigs produced and the size of the largest contig. Is this contig within the expected size range of the mitogenome of the species you are investigating? Is there a substantial difference between the reference, hybrid, and de novo assembly contig sizes? A comparison of the assembly statistics for our Triakidae mitogenomes is presented in Table 1 for reference.

**Table 1.** Summary of reference-based, hybrid, and de novo assembly statistics for five newly assembled Triakidae mitogenomes assembled using Ion Torrent sequence reads in bam format. # reads mapped, number of reads that mapped to the reference mitogenome (NC) or to each other; bp, base pairs; # of contigs, number of contigs created for hybrid and de novo assembly. Reference-based assembly was conducted in Geneious Prime v.2023.2 [19] and hybrid and de novo assembly were conducted using SPAdes v.3.15 [22].

Species	Reference assembly		Hybrid assembly		de novo assembly	
	**# reads mapped**	**Contig size (bp)**	**# of contigs**	**Size of largest contig (bp)**	**# of contigs**	**Size of largest contig (bp)**
*Galeorhinus galeus*	1,152	16,758	3	16,709	69,098	18,108
*Mustelus asterias*	3,193	16,763	2	16,928	192,700	13,356
*Mustelus mosis*	3,201	16,755	1	16,883	194,003	14,282
*Mustelus palumbes*	4,375	16,762	3	16,637	467,549	21,393
*Triakis megalopterus*	5,364	16,765	1	16,871	166,725	17,169

b. Sometimes, the de novo assembly may produce more than one contig for the mitogenome, which needs to be mapped together to produce a complete assembly as evident for *M. mosis* and *M. palumbes.* Very long de novo consensus sequences, like the one produced for *M. palumbes*, are often the result of large overlaps at each end. Circular consensus sequences can be produced by circularising de novo assemblies as described in Section B.

c. Align the three assemblies to each other using the Geneious alignment tool with default parameters.

d. Check the alignment for discrepancies in Geneious. If the assemblies are consistent with each other, save the alignment consensus sequence in fasta format for annotation in Section B. If there is a significant discrepancy between the three alignments (see Figure 3 as an example), further investigation is warranted, as described next.

**Figure 3.**
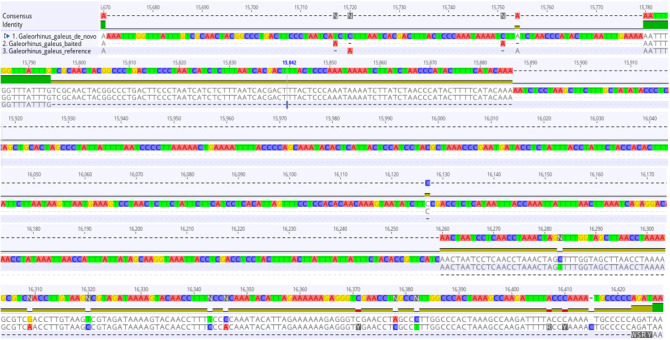
714-base pair mismatch region in the sequence alignment of the reference-based, hybrid (baited), and de novo mitogenome assemblies of *Galeorhinus galeus.* Green bars indicate matching regions in all assemblies, gold bars indicate matching regions in two of the assemblies, and black lines indicate alignment gaps.

**Figure 4.**
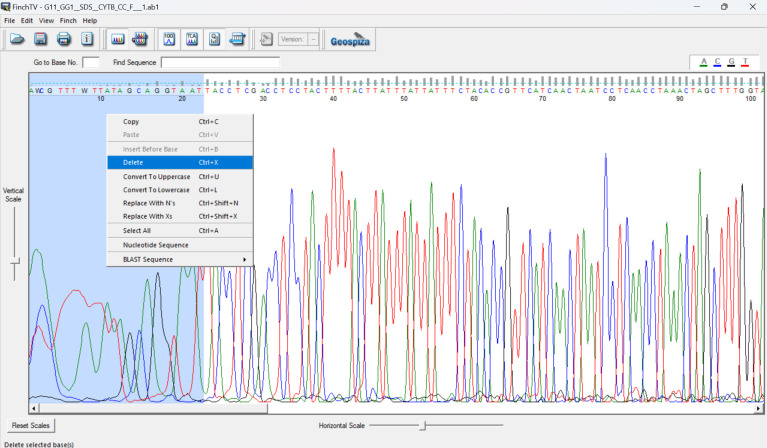
Trimming poor base calls from the ends of Sanger sequences in Finch TV 1.5 ( **https://digitalworldbiology.com/FinchTV** , **last accessed 1/12/2025).** The first and last ~20–30 nucleotides often show noisy peaks, where the base call is not definitive, and there are a few small, loopy peaks rather than tall, sharp peaks as seen from the 20th nucleotide to the 100th nucleotide. These bases must be removed from the sequence. Red peaks, thymine; green, adenine; blue, cytosine; black, guanine.

**Figure 5.**
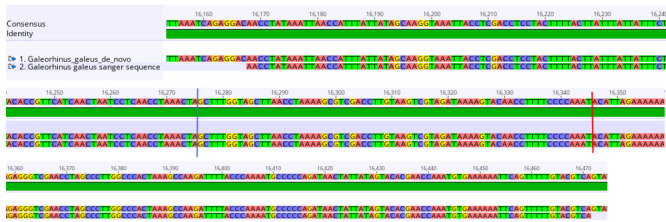
Sequence alignment of a section of the 714-base pair duplication in the de novo *Galeorhinus galeus* assembly (top sequence) and the sequence fragment obtained with Sanger sequencing (bottom sequence). The blue line marks the end of the duplicated cytochrome b (*Cytb)* section and the start of *tRNA^Thr’^* (thyronine). The red line marks the end of *tRNA^Thr’^* and the start of the *D-loop* (mitochondrial displacement loop).

**Figure 6.**
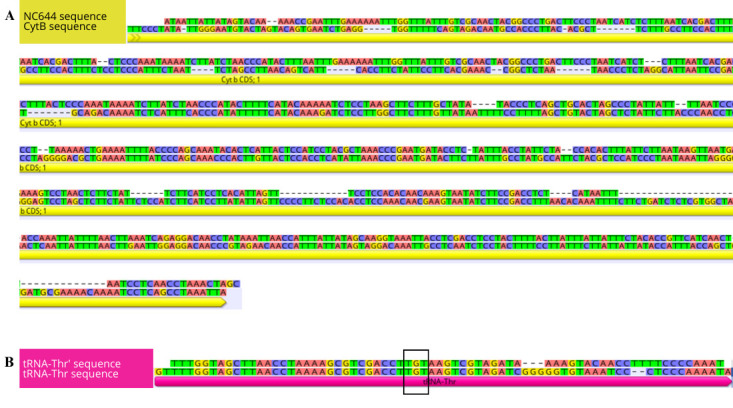
Sequence alignment of the duplication located between *tRNA^Pro^* (proline) and the *D-loop* (mitochondrial displacement loop) of *Galeorhinus galeus* with the *tRNA^Thr^* (thyronine) and cytochrome b (*Cytb*) genes. (A) Alignment of *NC_644_* and the partial *Cytb* gene. (B) Alignment of *tRNA^Thr’^* and the *tRNA^Thr^* gene. The black box indicates the anticodon sequences of the tRNAs.

5. As evident from Figure 3, a comparison of the assemblies for *G. galeus* revealed large gaps and base-pair mismatches in a section located before the control region, whereas alignment of our other mitogenome assemblies showed almost perfect agreement between the three different assemblies, with very few single base-pair mismatches. It is important to first ensure that you have high-quality sequences with good coverage of the mitogenome because this can also influence assembly quality. However, disagreement between reference, hybrid, and de novo assemblies can also occur when there is a large structural deviation in the mitogenome you are assembling that is missing from the reference mitogenome used for reference-based assembly. In order to investigate this further, we conducted Sanger sequencing on this region and compared the Sanger sequencing fragment to our three assemblies in Geneious. When there is a significant deviation in mitogenome structure compared to what is expected based on the mitogenomes of closely related species, it is good practice to confirm the anomaly before submitting annotated mitogenomes to GenBank.

a. Use a standard PCR and Sanger sequencing protocol, optimised to suit your primers, and extracted DNA samples to amplify and sequence the suspected duplication or rearrangement. We designed primers for *G. galeus* based on the de novo mitogenome assembly sequence to cover the duplicated region present in the assembly: *Cytb* CC F (5′-ACTTGAATTGGAGGGCAACC-3′) and *Dloop* Gga R (5′-AGGGTATGTGGGCCATATCA-3′).

b. After sequencing, manually trim the sequences in Finch TV (Figure 4) by selecting the noisy peaks at the beginning of the sequence (click on the first nucleotide, click and hold *ctrl + A*, click on the last nucleotide with a noisy peak, and then right-click and select *delete*). Do the same for the last ~20–30 nucleotides at the end of the sequence.

c. Align the trimmed sequences to the de novo, reference, and hybrid assemblies of the mitogenome under investigation in Geneious read mapper using the same approach described in step A4c above to confirm the presence of the duplication. Note whether the Sanger sequence matches with ~100% similarity to any of the assemblies. The alignment of the Sanger sequence fragment to the de novo assembly of *G. galeus* is shown in Figure 5. The Sanger sequence matches perfectly with this assembly but reveals a missing segment in the hybrid and reference assemblies, confirming the presence of the additional segment detected in the de novo assembly.

d. To confirm which portion of the mitogenome has undergone duplication, align the trimmed sequences with the reference genome assembly (which does not contain the duplication) using the Geneious read mapper with medium sensitivity settings and five iterations to predict which genes were duplicated (Figure 6). Annotation and functional investigation of the duplication are described in Section B.

e. Alignment of the duplicated region to the reference assembly revealed that the duplication is made up of a 646 bp non-coding (NC_646_) fragment of the cytochrome b (*Cytb*) gene at the 5′-end and a 68 bp *tRNA^Thr’^* at the 3′-end of the H-strand. This signifies a tandem duplication and random loss (TDRL) mutation, where the *Cytb* gene and *tRNA^Thr^* were duplicated, and the redundant paralogs underwent random deletion events to form non-coding regions that were then inserted into the control region [46,47]. Based on the TDRL and intramitochondrial recombination model, we speculate that *Cytb* and *tRNA^Thr^* underwent tandem duplication to form a *Cytb-tRNA^Thr^-Cytb’-tRNA^Thr’^* dimeric block [48]. Secondly, a random deletion occurred, resulting in the loss of redundant fragments of *Cytb’* and *tRNA^Thr^’* and consequently a loss in function of these genes. Lastly, the remnants of the duplication recombined into the *D-loop*. To view the location of the duplication in the mitogenome, see Figure 8 in Section B below.

f. The reference-based assembly was unable to assemble the duplication and collapsed all similar reads together. Reference assembly is biased by the structure of the reference mitogenome used, which makes it unsuitable for detecting structural deviations when assembling short reads. This is easier to avoid when longer PacBio and ONT reads are used for assembly to a reference mitogenome. The hybrid assembly approach assembled a portion of the duplicated region but not all of it. Only de novo assembly was able to properly assemble the duplication, as confirmed by the Sanger sequences. In this case, we proceeded with the de novo assembly consensus sequence (exported in fasta format) and used it for mitogenome annotation in Section B.

**B. Mitogenome annotation**

In this section, we present a pipeline to annotate and characterise mitogenome features for comparison of newly assembled mitogenomes to other mitogenomes of closely related species. Calculations are performed using default settings unless otherwise stated; when applicable, make sure to select the correct genetic code for your species. In our case, we used the vertebrate mitochondrial setting (Table 2). The programs give multiple data output files. We present the tables and figures we created for *Galeorhinus galeus* as an example using this data, but there are other statistics to explore too (consult the manuals linked throughout the protocol).

**Figure 7.**
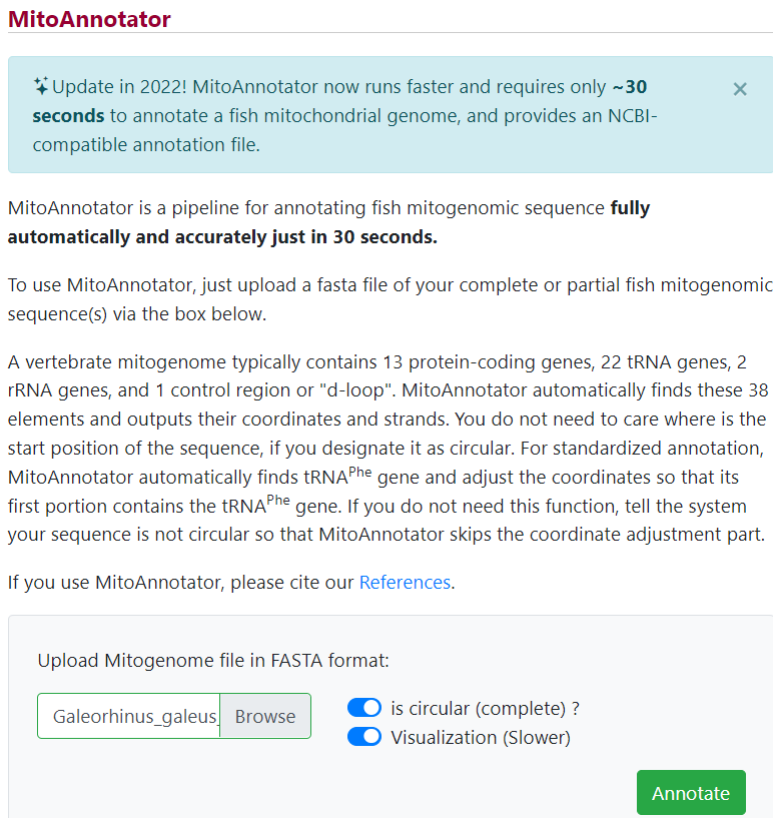
Annotating mitogenome consensus sequences in MitoAnnotator in MitoFish v.3.72 webserver ( http://mitofish.aori.u-tokyo.ac.jp/annotation/input/ , last accessed 1/12/2025) [8,40]

**Figure 8.**
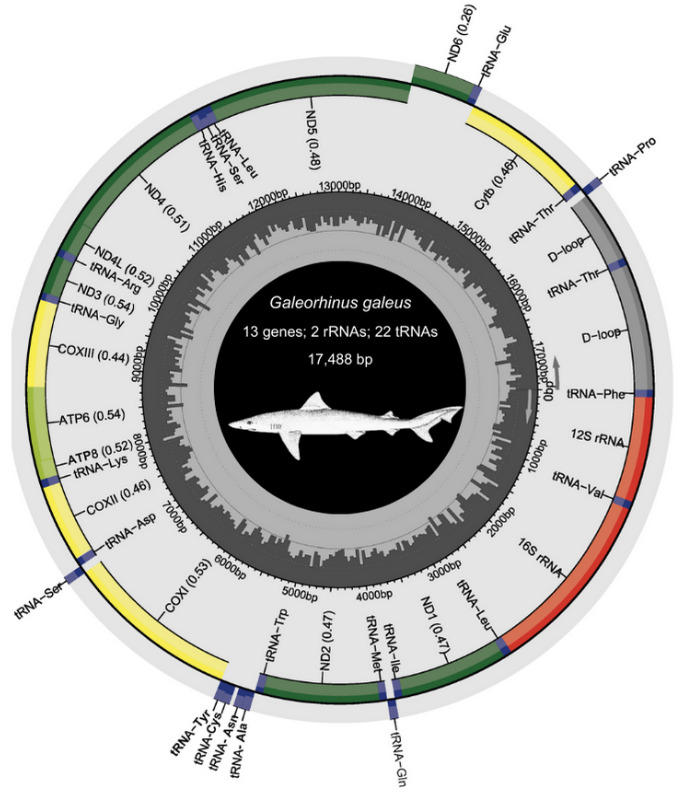
Annotated mitogenome of *Galeorhinus galeus* (Linnaeus 1758) generated with MitoAnnotator in MitoFish v.3.72 webserver ( http://mitofish.aori.u-tokyo.ac.jp/annotation/input/, last accessed 1/12/2025) [34,35]. In the outermost ring, the inner genes are transcribed clockwise, while the outer genes are transcribed counterclockwise. The middle grey circle shows the GC content. The inner number indicates base pairs (bp). The species sketch was retrieved from the Food and Agriculture Organization (FAO) website (www.fishbase.se, last accessed 1/12/2025).

**Figure 9.**
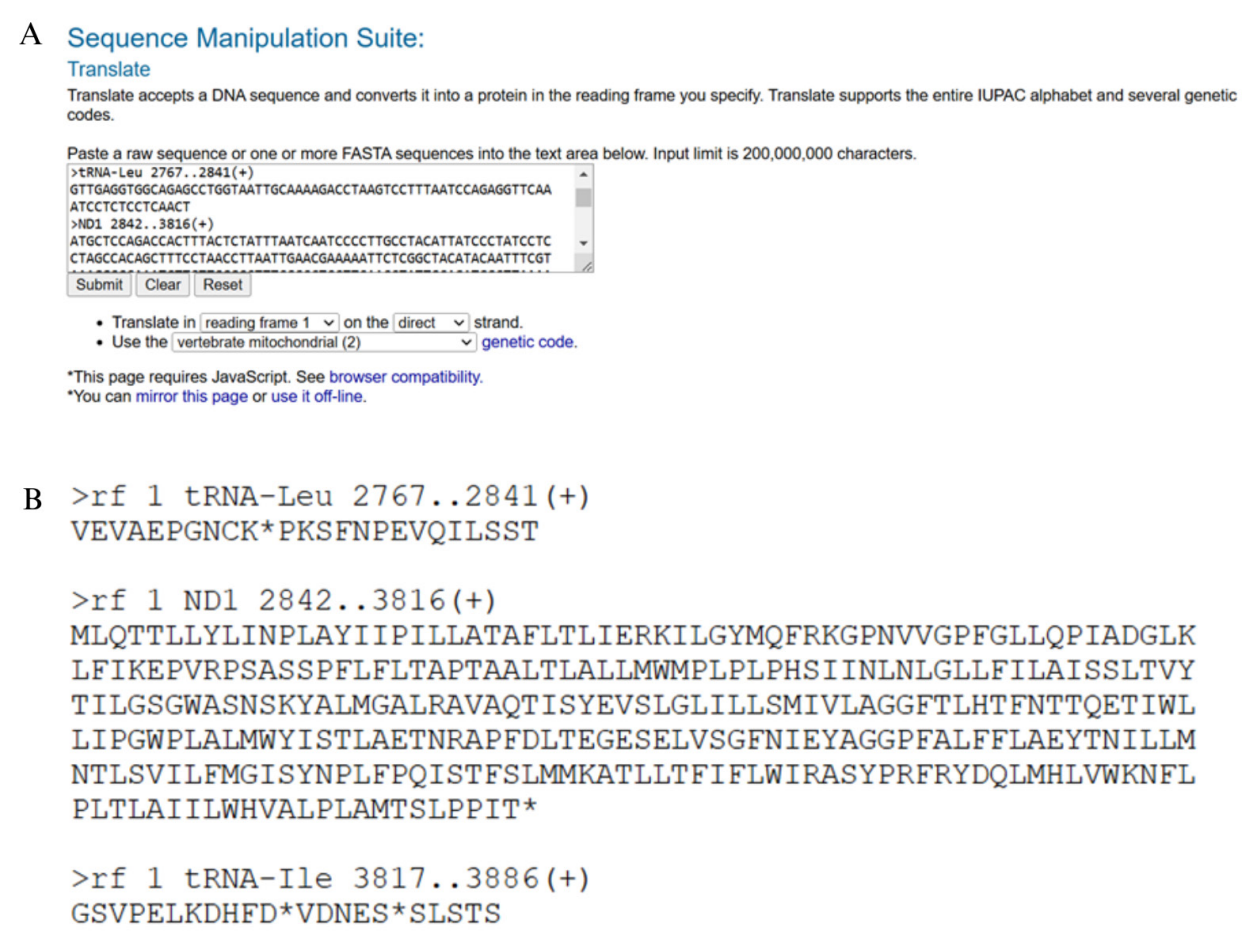
Checking the reading frame of protein coding genes in Sequence Manipulation Suite 2 ( https://www.bioinformatics.org/sms2/translate.html , last accessed 1/12/2025) [36]. The input file containing the nucleotide sequence of each protein-coding gene from MitoAnnotator is pasted into the translation box (A) and produces an output window with the amino acid code for each gene (B). Protein-coding genes can be checked for internal stop codons, which may indicate a reading frame shift and error in the consensus sequence during mitogenome assembly. Remember to select the correct genetic code for your species (vertebrate mitochondrial Table 2 was selected here).

**Figure 10.**
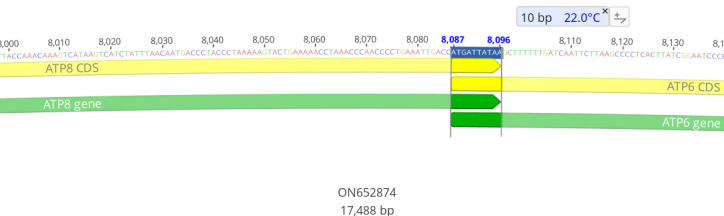
An example of a 10-base pair overlap between ATP8 and ATP6 in the mitogenome of *Galeorhinus galeus*

1. Open MitoAnnotator on your preferred web browser (http://mitofish.aori.u-tokyo.ac.jp/annotation/input/, last accessed 1/12/2025). Select your mitogenome consensus sequence (saved in fasta format in Section A) as the input file and click *Annotate* (Figure 7).

a. Download and save the zip folder produced by MitoAnnotator containing the annotated mitogenome, gene sequences in fasta format, and the annotated mitogenome in GenBank format. Edit and enlarge gene names with a PDF editor of choice and include protein-coding genes (PCGs), transfer (t)RNA and ribosomal (r)RNA counts, the total mitogenome length, and species-specific images [ours were obtained from the Food and Agriculture Organization website (www.fishbase.se, last accessed 1/12/2025)] (see Figure 8).

b. Open the gene feature file containing separate sequences for all PCGs, rRNAs, and tRNAs in one file (.FA file with the extension *_genes*) in the MitoAnnotator folder saved in step B1a above, copy and paste its contents into Sequence Manipulation Suite 2, and select the correct genetic code for your species. The output window generated shows the translated version of each gene. Check that the reading frame is correct for each PCG (Figure 9). There should be no internal stop codons (indicated by asterisks). The number of nucleotides in each PCG should be a multiple of three for translation to occur correctly. Gaps in the alignment can cause reading frame shifts, resulting in stop codons within coding sequences. You would expect to find stop codons in the tRNAs and rRNAs because they do not contain three-letter codons. If there are stop codons within the PCG sequences, go back to the assembly alignment in step A4d and check to see whether ambiguities in the consensus sequence need to be edited. If this is the case, the edited consensus sequence will need to be reannotated in MitoAnnotator and checked again in Sequence Manipulation Suite to ensure reading frame shifts have been corrected before proceeding to the next step.

c. Import the GenBank file generated by MitoAnnotator (available in the zip folder downloaded in step A1a above) into Geneious and check the annotated sequences to ensure completeness and manually count overlapping regions and intergenic spaces between PCGs, rRNAs, tRNAs, and non-coding regions (Figure 10).

2. After confirming that each region of the mitogenome has been correctly annotated, submit the GenBank file to GenBank by following the guidelines for submission using BankIt (https://www.ncbi.nlm.nih.gov/WebSub/, last accessed 1/12/2025).

3. Calculate mitogenome statistics (Table 2).

a. Calculate A+T and G+C content of each separate PCG, all tRNAs, and the whole mitogenome in DAMBE. Use the fasta file *Galeorhinus_galeus_genes.fa* generated by MitoAnnotator as the input file. Click *Seq. Analysis|Nucleotide & di-nuc Frequency*. Place the gene of interest into the input file panel to calculate statistics separately or put them all into the input panel to calculate them together.

b. Calculate base composition skewness from the nucleotide composition results with the following formula: AT-skew = [A - T]/[A + T] and GC-skew = [G - C]/[G + C] [49].

**Table 2.** Nucleotide composition and skewness values for the mitogenome genes of *Galeorhinus galeus* calculated in DAMBE v.7.0.35 [38,39]. A: number of adenine nucleotides; C: cytosine; G: guanine; T: thymine. AT-content: percentage of A and T nucleotides relative to the total number of nucleotides in the mitogenome sequence; GC-content: percentage of G and C nucleotides. Base composition skewness was calculated from the nucleotide composition results using the following formula: AT-skew = [A - T]/[A + T] and GC-skew = [G - C]/[G + C] [49]. The highest positive and lowest negative values are indicated in bold.

Gene	A	C	G	T	AT-content	GC-content	AT-skew	GC-skew
12S rRNA	309	214	189	240	57.66807	42.33193	0.125683	-0.06203
16S rRNA	598	329	286	454	63.10738	36.89262	**0.136882**	-0.06992
ND1	290	260	119	306	61.12821	38.87179	-0.02685	-0.37203
ND2	325	306	100	314	61.14833	38.85167	0.017214	-0.50739
COX1	425	351	249	532	61.46435	38.53565	-0.11181	-0.17
COX2	223	164	102	202	61.50507	38.49493	0.049412	-0.23308
ATP8	62	39	12	55	69.64286	30.35714	0.059829	**-0.52941**
ATP6	200	168	76	239	64.27526	35.72474	-0.08884	-0.37705
COX3	223	203	124	236	58.39695	41.60305	-0.02832	-0.24159
ND3	84	99	53	113	56.44699	43.55301	-0.14721	-0.30263
ND4L	73	88	38	98	57.57576	42.42424	-0.1462	-0.39683
ND4	410	375	157	438	61.44928	38.55072	-0.03302	-0.40977
ND5	578	476	189	587	63.6612	36.3388	-0.00773	-0.43158
ND6	104	48	159	211	60.34483	39.65517	**-0.33968**	**0.536232**
CYTB	306	299	145	395	61.22271	38.77729	-0.12696	-0.34685
D-LOOP	619	410	200	626	67.1159	32.8841	-0.00562	-0.34426
tRNAs	502	290	338	493	61.30622	38.69378	0.009045	0.076433
Whole genome	5444	4278	2366	5400	62.00823	37.99177	0.004058	-0.28778

c. Construct graphs for nucleotide composition and skewness in R (Figure S1). Use Table 2 as the input data.

# Path to output PDFs

pdfPath <- './Nucleotide Composition Plot/'

# Import the data

library(readxl)

G_galeus <- read_excel("./Nucleotide Composition Plot/Nucleotide Composition.xlsx", sheet = "Galeorhinus galeus")

# Initialise PDF

pdf(paste(pdfPath, '/Nucleotide_Composition.pdf', sep=''), width=8, height=6)

library(ggplot2)

# G_galeus: Nucleotide composition

ggplot(G_galeus) +

 geom_line(aes(x=Gene, y=AT_content, group=1), color = "coral", size=1) +

 geom_line(aes(x=Gene, y=GC_content, group=1), color = "darkturquoise", size=1) +

 labs(title = "Galeorhinus galeus", x="Gene", y="Content(%)") +

 theme_minimal() +

 theme(plot.title = element_text(hjust=0.5, size=18, face='italic')) +

 theme(axis.text.x = element_text(angle = 52, size=9))

# G_galeus: Nucleotide skewness

ggplot(G_galeus) +

 geom_line(aes(x=Gene, y=AT_skew, group=1), color = "coral", size=1) +

 geom_line(aes(x=Gene, y=GC_skew, group=1), color = "darkturquoise", size=1) +

 labs(title="Galeorhinus galeus", x="Gene", y="Skewness") +

 theme_minimal() +

 theme(plot.title = element_text(hjust=0.5, size=18, face='italic')) +

 theme(axis.text.x = element_text(angle = 52, size=9))

.=dev.off()

d. Calculate the amino acid composition and relative synonymous codon usage (RSCU) of all PCGs in DAMBE (Table 3). RSCU is the ratio of the observed frequency of codons to the expected frequency given that all the synonymous codons for the same amino acids are used equally. Use the complete mitogenome fasta file generated by MitoAnnotator as the input file. Click *Seq. Analysis|Codon Usage|Relative synonymous codon usage*. Place all PCGs in the right-hand panel and click *Run*.

**Table 3.** Total amino acid counts and relative synonymous codon usage (RSCU) for the mitogenome genes of *Galeorhinus galeus* calculated in DAMBE v.7.0.35 [38,39]. RSCU is the ratio of the observed frequency of codons to the expected frequency given that all the synonymous codons for the same amino acids are used equally. The highest and lowest RSCU values are indicated in bold. Codon: one letter codon code; Codon2: three letter codon code; N: number of occurrences of the codon in the mitogenome; %: percentage occurrences of the codon out of all codon occurrences; Fraction: proportion of a specific codon out of all codons for a specific amino acid.

**Codon**	**Codon2**	**Amino acid**	**N**	%	**RSCU**	**Fraction**
A	GCU	Ala	74	1.947881	1.053	0.263316
B	GCG	Ala	2	0.052645	**0.028**	0.007002
C	GCC	Ala	104	2.737563	1.48	0.370093
D	GCA	Ala	101	2.658594	1.438	0.35959
A	UGU	Cys	13	0.342195	1.083	0.5415
B	UGC	Cys	11	0.28955	0.917	0.4585
A	GAU	Asp	34	0.894972	0.958	0.479
B	GAC	Asp	37	0.973941	1.042	0.521
A	GAG	Glu	12	0.315873	0.245	0.1225
B	GAA	Glu	86	2.263754	1.755	0.8775
A	UUU	Phe	145	3.816794	1.203	0.6015
B	UUC	Phe	96	2.526981	0.797	0.3985
A	GGU	Gly	52	1.368781	0.908	0.227
B	GGG	Gly	23	0.605422	0.402	0.1005
C	GGC	Gly	65	1.710977	1.135	0.242004
D	GGA	Gly	89	2.342722	1.555	0.31294
A	CAC	His	56	1.474072	1.098	0.549
B	CAU	His	46	1.210845	0.902	0.451
A	AUU	Ile	239	6.291129	1.414	0.707
B	AUC	Ile	99	2.605949	0.586	0.293
A	AAA	Lys	81	2.13214	1.952	0.976
B	AAG	Lys	2	0.052645	0.048	0.024
A	CUA	Leu1	173	4.55383	1.58	0.263289
B	CUC	Leu1	95	2.500658	0.868	0.144643
C	CUG	Leu1	19	0.500132	0.174	0.028995
D	CUU	Leu1	151	3.97473	1.379	0.229795
A	UUA	Leu2	204	5.369834	1.838	0.306282
B	UUG	Leu2	18	0.473809	0.162	0.026996
A	AUG	Met	36	0.947618	0.444	0.222
B	AUA	Met	126	3.316662	1.556	0.778
A	AAC	Asn	85	2.237431	1.118	0.559
B	AAU	Asn	67	1.763622	0.882	0.441
A	CCU	Pro	53	1.395104	1.005	0.251187
B	CCG	Pro	3	0.078968	0.057	0.014246
C	CCC	Pro	57	1.500395	1.081	0.270182
D	CCA	Pro	98	2.579626	1.858	0.464384
A	CAA	Gln	85	2.237431	1.789	0.8945
B	CAG	Gln	10	0.263227	0.211	0.1055
A	CGA	Arg	37	0.973941	**2.027**	0.50675
B	CGC	Arg	16	0.421163	0.877	0.21925
C	CGG	Arg	6	0.157936	0.329	0.08225
D	CGU	Arg	14	0.368518	0.767	0.19175
A	AGC	Ser1	33	0.86865	1.245	0.2075
B	AGU	Ser1	20	0.526454	0.755	0.125833
A	UCA	Ser2	98	2.579626	1.798	0.299667
B	UCC	Ser2	59	1.55304	1.083	0.1805
C	UCG	Ser2	4	0.105291	0.073	0.012167
D	UCU	Ser2	57	1.500395	1.046	0.174333
A	ACA	Thr	125	3.29034	1.773	0.44325
B	ACU	Thr	77	2.026849	1.092	0.273
C	ACC	Thr	75	1.974204	1.064	0.266
D	ACG	Thr	5	0.131614	0.071	0.01775
A	GUU	Val	62	1.632008	1.363	0.34075
B	GUG	Val	11	0.28955	0.242	0.0605
C	GUC	Val	30	0.789681	0.659	0.16475
D	GUA	Val	79	2.079495	1.736	0.434
A	UGA	Trp	112	2.948144	1.851	0.9255
B	UGG	Trp	9	0.236904	0.149	0.0745
A	UAC	Tyr	47	1.237168	0.764	0.382
B	UAU	Tyr	76	2.000526	1.236	0.618

e. Construct amino acid composition and RSCU plots in R using Table 3 as the input data (Figure S2).

# Path to output PDFs

pdfPath <- './Codon Usage'

# Import the data

library(readxl)

G_galeus <- read_excel("./Codon Usage/Codon Usage.xlsx", sheet = "Galeorhinus galeus")

# Initialise PDF

pdf(paste(pdfPath, '/AA_composition.pdf', sep=''), width=8, height=6)

library(ggplot2)

# G_galeus: Amino acid composition

ggplot(G_galeus, aes(x = Amino_Acid, y = N)) +

 geom_bar(stat = "identity", fill = 4) +

 theme_minimal() +

 theme(plot.title = element_text(hjust=0.5, size=20, face='italic'))

# G_galeus : RSCU

ggplot(G_galeus, aes(x = Amino_Acid, y=RSCU, fill=Codon, label=Codon2)) +

 geom_bar(stat = "identity") + geom_text(size = 3, position = position_stack(vjust = 0.5)) +

 theme_minimal() +

 theme(plot.title = element_text(hjust=0.5, size=20, face='italic')) +

 theme(legend.position="none") +

 scale_fill_manual('Codon', values=c('#FFA54F', '#00C1AA', '#FF69B4', '#9370DB'))

.=dev.off()

4. Use the complete mitogenome sequence in fasta format as the input file, search for mammalian mitochondrial tRNA genes (*-mtmam*), and predict tRNA secondary structure using the mammalian mitochondrial genetic code (*-gcmam*) in the ARWEN v.1.2.3 webserver [40] (http://130.235.244.92/ARWEN/, last accessed 1/12/2025). Alternatively, select the correct genetic code for your study organism. Note that ARWEN is specifically designed for metazoan mitochondrial genomes. Investigate other software for plant mitogenomes and plastids (e.g., MFannot [50] and Mitofy [51]). tRNA annotation can also be conducted using the tRNAscanSE webserver v.2.0 [41] (http://lowelab.ucsc.edu/cgi-bin/tRNAscan-SE2.cgi, last accessed 1/12/2025). We selected the sequence source as *vertebrate mitochondrial* and uploaded the input mitogenome file in fasta format.

a. MXfold2 [42] can be used to verify the secondary structure of duplicated tRNAs. We used it to compare the secondary structure of the duplicated *tRNA^Thr’^* to that of *tRNA^Thr^* in *Galeorhinus galeus* (Figure S3). Simply navigate to the MXfold2 server (http://ws.sato-lab.org/mxfold2/, last accessed 1/12/2025), paste your tRNA sequence in fasta format into the search box, and click *Predict*.

**[Tip 1]** tRNA-Scan has been trained using a broad set of vertebrate mitochondrial tRNAs. However, because it is an algorithmic model, rich parameterisation can cause overfitting to the training data, preventing robust predictions [52]. MXFold2 is a hybrid method that integrates folding scores calculated by a deep neural network trained with a large quantity of data but avoids overfitting rich-parameterised weight parameters to the training data by resorting to the thermodynamic parameters for assessing previously unobserved substructures [42]. MXfold2 produces a less stable secondary structure for *tRNA^Thr’^* than for *tRNA^Thr^*.

5. Characterisation of the control region using Tandem Repeat Finder (https://tandem.bu.edu/trf/trf.html, last accessed 1/12/2025)

a. Navigate to the Tandem Repeat Finder webpage and click *Submit Sequence* in the top menu bar. Select the *Basic Options* to run parameters on default. If you want to modify a specific parameter, select *Advanced Options*. Select your mitogenome consensus sequence as the input and click *Submit sequence*. Click on the *Tandem Repeats Report* to access the information presented in Table 4.

6. Compile the statistics calculated from the various mitogenome characterisation techniques described in Section B into a summary table to compare the features of your mitogenomes with other closely related species (Table 5). Gene positions and sizes (in bp) can be obtained from GenBank files generated during the submission process.

## Validation of protocol

This full protocol has been used and validated in the following research article:

• Winn et al. [56]. A comprehensive phylogenomic study unveils evolutionary patterns and challenges in the mitochondrial genomes of Carcharhiniformes: A focus on Triakidae. *Genomics*. 116(1): 110771. doi: 10.1016/j.ygeno.2023.110771

In summary, a total of 38,889,488 unpaired raw reads with an average of 315 bp per read were generated from the whole genome shotgun sequencing of five triakid species with the Ion GeneStudio^TM^ S5 Prime System (available as a BioProject on the SRA database; PRJNA997468). Although sequencing output was generated for the whole genome, we only assembled the mitogenomes for this study (filtered mitogenome reads are available as Data 1–5 on Dryad; doi: 10.5061/dryad.sj3tx969h ). Mitogenome assembly was conducted using the three-step approach described in Section A: mapping reads to the reference mitogenome of a closely related species (*Mustelus mustelus*, NC 039629) in Geneious, extracting the reads that mapped to the reference and assembling them de novo in SPAdes, and assembling the reads entirely de novo. The number of reads that mapped to the reference mitogenome, contig sizes, and total number of contigs produced in the de novo assemblies are shown in Table 1. A 714 base pair duplication between the control region and *tRNA^Pro^* was detected in the mitogenome of *Galeorhinus galeus*, which was determined to be a tandem duplication repeat of the *Cytb* gene and *tRNA^Thr^*. The five newly assembled mitogenomes were then annotated as described in Section B, and the functionality of the duplicated *tRNA^Thr^* for *G. galeus* was explored in depth using different tRNA folding models (see Figure S3 and Figure 4 in the original research article). It was found that the duplicated *tRNA^Thr^* was likely non-functional. Mitogenome sequence files for our Triakidae mitogenomes are available on GenBank (ON075075, ON075076, ON075077, ON652873, and ON652874) or Dryad (Data 8–12).

**Table 4.** Description of tandem repeats found in the control region of the Triakidae mitogenomes

Species	Control region limits	Indices	Period Size	Copy Number	Consensus Size	Matches (%)	Indels (%)	Score	A	C	G	T	Entropy (0-2)
*Galeorhinus galeus*	15,633–16,754	24--239	108	2.0	108	100	0	432	30	25	8	36	1.85
		938--1032	46	2.1	46	82	4	111	31	24	5	38	1.77
*Mustelus asterias*	15,641–16,762	65--102	17	2.2	17	86	13	51	42	18	7	31	1.79
		61--106	18	2.5	19	80	20	53	43	19	6	30	1.76
		131--221	46	1.9	47	86	4	130	31	27	4	36	1.77
*Mustelus manazo*	15,640–16,707	65-102	17	2,2	17	86	13	51	42	18	7	31	1,79
		61-106	18	2,5	19	80	20	53	43	19	6	30	1,76
*Mustelus palumbes*	15,641–16,762	65--102	17	2.2	17	86	13	51	42	18	7	31	1.79
		61--106	18	2.5	19	80	20	53	43	19	6	30	1.76
		131--221	46	1.9	47	84	4	121	31	26	4	37	1.76
*Triakis megalopterus*	15,699–16,765	66--103	17	2.2	17	86	13	51	42	18	7	31	1.79
		62--107	18	2.5	19	80	20	53	43	19	6	30	1.76
*Note: No tandem repeats were found in the control region of Hemitriakis japanica, Mustelus griseus, Mustelus mosis, or Mustelus mustelus.*													
The summary table includes the following information:													
1. Indices of the repeat relative to the start of the sequence.													
2. Period size of the repeat.													
3. Number of copies aligned with the consensus pattern.													
4. Size of consensus pattern (may differ slightly from the period size).													
5. Percentage of matches between adjacent copies overall.													
6. Percentage of indels between adjacent copies overall.													
7. Alignment score.													
8. Percentage composition for each of the four nucleotides.													
9. Entropy measure based on percent composition.													

**Table 5.** Features of the complete mitochondrial genome of *Galeorhinus galeus*. IGN values represent intergenic nucleotides and overlapping nucleotides (−). Start, start codon; Stop, stop codons; H-strand, heavy strand; L-strand, low strand; bp, base pairs.

Gene (anticodon)	Position		Strand	Size	Codon		IGN
	**Start**	**End**		**Nucleotide (bp)**	**Start**	**Stop**	
tRNA-*Phe* (GAA)	1	69	H	69			0
12S rRNA	70	1021	H	952			0
tRNA-*Val* (TAC)	1022	1093	H	72			0
16S rRNA	1094	2760	H	1667			0
tRNA-*Leu*1 (TAA)	2761	2835	H	75			0
ND1	2836	3810	H	975	ATG	TAA	0
tRNA-*Ile* (GAT)	3811	3880	H	70			0
tRNA-*Gln* (TTG)	3879	3950	L	72			-2
tRNA-*Met* (CAT)	3951	4019	H	69			0
ND2	4020	5064	H	1045	ATG	T	0
tRNA-*Trp* (TCA)	5065	5135	H	71			0
tRNA-*Ala* (TGC)	5137	5205	L	69			1
tRNA-*Asn* (GTT)	5206	5278	L	73			0
OL	5279	5313	–	35			0
tRNA-*Cys* (GCA)	5314	5382	L	69			0
tRNA-*Tyr* (GTA)	5384	5453	L	70			1
COI	5455	7011	H	1557	GTG	TAA	1
tRNA-*Ser*1 (TGA)	7012	7082	L	71			0
tRNA-*Asp* (GTC)	7086	7155	H	70			3
COII	7163	7853	H	691	ATG	T	7
tRNA-*Lys* (TTT)	7854	7927	H	74			0
ATP8	7929	8096	H	168	ATG	TAA	1
ATP6	8087	8769	H	683	ATG	TA	-10
COIII	8770	9555	H	786	ATG	TAA	0
tRNA-*Gly* (TCC)	9558	9627	H	70			2
ND3	9628	9976	H	349	ATG	T	0
tRNA-*Arg* (TCG)	9977	10046	H	70			0
ND4L	10047	10343	H	297	ATG	TAA	0
ND4	10337	11717	H	1381	ATG	T	-7
tRNA-*His* (GTG)	11718	11786	H	69			0
tRNA-*Ser*2 (GCT)	11787	11853	H	67			0
tRNA-*Leu*2 (TAG)	11854	11925	H	72			0
ND5	11926	13755	H	1830	ATG	TAA	0
ND6	13751	14272	L	522	ATG	AGG	-5
tRNA-*Glu* (TTC)	14273	14342	L	70			0
Cytb	14345	15489	H	1145	ATG	TA	2
tRNA-*Thr* (TGT)	15490	15561	H	72			0
tRNA-*Pro* (TGG)	15564	15632	L	69			2
Control region (CR)	16347	17488	H	1142			714

## General notes and troubleshooting

**General notes**

The mitogenome is a small portion of an organism’s genomic material that is easy to obtain and characterise. Nevertheless, harnessing the full potential of this molecular tool necessitates a meticulous approach to assembling and characterising each protein-coding gene, tRNA, rRNA, and even non-coding regions like the control region. Our bioinformatic pipeline serves as a comprehensive guide to facilitate the successful execution of these actions.

1. Detecting structural deviations in the mitogenome

Section A covers the assembly procedure necessary to reveal deviations from the expected mitogenome structure, reducing computational requirements while mitigating the bias that may arise from reference-based assembly alone by incorporating the hybrid assembly technique. Table 1 shows how the hybrid assembly method resulted in complete contigs that differed in size from the reference assembly. This is largely due to the high variability of the control region, which can contain very different repeat patterns and numbers, even in closely related species [53–55]. The hybrid assembly technique allows for these differences to be detected. We suggest starting with reference-based and hybrid assembly and comparing these two assemblies to each other if you want to avoid the computational requirements of de novo assembly. If there are large discrepancies between the two assemblies as demonstrated in Figure 3, then de novo assembly should be used to investigate the full extent of structural change. It is important to note that complex multi-copy duplications may not be detected when assembling short reads de novo and long-read technologies would be valuable in this regard. Annotate the de novo assembly in MitoAnnotator as described in Section B; if the annotation results in a complete mitogenome sequence with a structural deviation, sequence the region of interest to confirm its presence. Use a high-quality assembly as the reference mitogenome and make sure it is as closely related to your study species as possible.

2. Comparative mitogenomics

Section B details an annotation pipeline that can be followed to characterise and compare new mitogenomes to other closely related mitogenomes. Figures S1 and S2 are examples of nucleotide composition and relative synonymous codon usage plots for a mitogenome. These plots can be generated for newly assembled mitogenomes and publicly available mitogenomes of closely related species, as well as those of more distantly related species. Look for large deviations in AT/GC content/skewness and RSCU for each gene in your mitogenomes and compare these statistics to the mitogenomes of both closely and more distantly related species. Always confirm first whether there is an annotation or calculation error before drawing any conclusions regarding unusual mitogenome patterns.

**Troubleshooting**

**Problem 1:** Disagreement between reference-based, hybrid, and de novo alignments.

**Possible causes:** The reference mitogenome is of low quality or too distantly related to the mitogenome you are assembling.

If this is not the case, there may be a deviation in structure from the reference genome.

**Solution:** Select a high-quality assembly as the reference mitogenome and make sure it is as closely related to your sequenced mitogenome as possible. If there is still disagreement between the three assembly approaches, annotate the de novo assembly. If annotation results in a complete mitogenome sequence with a structural deviation such as a duplication or a deletion, sequence the region of interest to confirm its presence (Section A).

**Problem 2:** Mitogenome annotation yields unusual signatures (significant deviations from closely related mitogenomes).

**Possible causes:** The reading frames for a PCG are incorrect, or the genes are not in the correct orientation. If this is not the case, there may be a deviation in structure from the reference genome.

**Solution:** Check the assemblies in Geneious, correct reading frame errors, and make sure gene orientation is correct in alignments and input files for DAMBE. Sequence and confirm structural deviations (Section A).

## Supplementary information

The following supporting information can be downloaded here:

1. Figure S1. Nucleotide composition (A) and skewness (B) plots for the mitogenome of *Galeorhinus galeus* constructed using ggplot2 in R Studio (https://posit.co/products/open-source/rstudio/).

2. Figure S2. Amino acid composition (A) and relative synonymous codon usage (B) plots for the mitogenome of *Galeorhinus galeus* constructed using ggplot2 in R Studio (https://posit.co/products/open-source/rstudio/).

3. Figure S3. Annotation and folding prediction of tRNA^Thr’^ (thyronine) (A) and tRNA^Thr^ (B) in *Galeorhinus galeus* with the MXfold2 webserver [42] (http://ws.sato-lab.org/mxfold2/).
